# Genome-Wide Identification and Expression Analysis of the Hsp70 Gene Family in *Hylocereus undatus* Seedlings Under Heat Shock Stress

**DOI:** 10.3390/ijms27020816

**Published:** 2026-01-14

**Authors:** Youjie Liu, Ke Wen, Hanyao Zhang, Xiuqing Wei, Liang Li, Ping Zhou, Yajun Tang, Dong Yu, Yueming Xiong, Jiahui Xu

**Affiliations:** 1 Fruit Science Institute, Fujian Academy of Agricultural Sciences, Fuzhou 350013, China; liuyoujie@faas.cn (Y.L.); weixiuqing@faas.cn (X.W.); liliang@faas.cn (L.L.); zhouping@faas.cn (P.Z.); yajun_t@163.com (Y.T.); yudong@faas.cn (D.Y.); xiongyueming@faas.cn (Y.X.); 2 Key Laboratory for Forest Resources Conservation and Utilization in the Southwest Mountains of China, Ministry of Education, Southwest Forestry University, Kunming 650224, China; wenke@swfu.edu.cn (K.W.); zhanghanyao@swfu.edu.cn (H.Z.)

**Keywords:** *Hylocereus undatus*, Hsp70 gene family, genome evolution, heat stress expression patterns, key candidate gene *HuHsp70-11*

## Abstract

*Hylocereus undatus* growth is limited by long-term heat stress, and heat shock protein 70 (Hsp70) is crucial in the plant’s heat stress (HS) response. In a previous study, transcriptomic data revealed that Hsp70 family members in pitaya seedlings respond to temperature changes. This study identified 27 *HuHsp70* genes in pitaya, analyzed their physicochemical properties (such as molecular weight and isoelectric point), and divided them into five subfamilies with conserved gene structures, motifs (short conserved sequence patterns), and cis-acting elements (regulatory DNA sequences). The Ks value (synonymous substitution rate) ranged from 0.93~3.54, and gene duplication events occurred between 71.17 and 272.19 million years ago (Mya). Under HS, eight and nine differentially expressed genes (DEGs) were detected at 24 h and 48 h, respectively. Quantitative real-time PCR (qRT-PCR, a method for measuring gene expression) verified the expression trends, with HuHsp70-11 expression increasing with heat shock duration, indicating that *HuHsp70-11* is a key candidate. Gene Ontology (GO) and Kyoto Encyclopedia of Genes and Genomes (KEGG) analyses revealed that *HuHsp70s*, especially *HuHsp70-11*, play key roles in responding to high temperatures (HT) in *H. undatus* seedlings. A potential model by which *HuHsp70-11* removes excess reactive oxygen species (ROS) and enhances cell membrane permeability was constructed. These results provide new perspectives for exploring the HS response mechanisms and adaptability of *H. undatus* plants to heat stress.

## 1. Introduction

In recent years, the greenhouse effect has led to an increase in the frequency of extreme weather events, and high temperatures have become a significant limiting environmental factor affecting plant growth, development, yield, and quality [1,2]. This issue is particularly prominent for pitaya (*Hylocereus* spp.), a globally important tropical fruit that has seen rapid expansion—by 2024, its global planting area had reached 1.5 million hectares with an annual output of approximately 10 million tons, dominated by producers like China, Vietnam, and Thailand. Heat stress (HS) causes a series of adverse reactions in plants, including effects on cell membrane structure, the antioxidant system, osmotic regulation, phytohormone regulation, and high-temperature response genes, which ultimately have a significant impact on photosynthesis in plants [3,4,5]. For pitaya, HS severely inhibits photosynthetic efficiency, pollen viability, and fruit development; in extreme cases, greenhouse temperatures exceeding 60 °C can directly scorch developing fruits, leading to premature rot and substantial yield losses. To better survive and reproduce, plants have evolved various physiological, cellular, and molecular mechanisms that are involved in resistance to HS [6,7]. Currently, many genes and proteins associated with HS in plants have been identified, including signaling components (protein kinases and transcription factors) and functional genes (heat shock proteins and catalases) [5]. When HS occurs, cells are protected from damage by regulating the expression of these genes and participating in the folding and degradation of proteins. Therefore, a rapid response during this process is crucial for the tolerance of HS [8].

The most critical response of HS is the induction of heat shock protein (Hsp). The process can be divided into the activation of heat shock transcription factor (Hsf), the binding of HSF to heat shock elements (Hse), and the transcription of the Hsp gene, which ultimately binds to the Hsp gene through molecular chaperones. During this period, the expression of some normal genes in cells is inhibited [7,9,10]. Hsp has a molecular weight between 15 and 110 kDa and is located in different cell structures. They are divided into the following families according to their molecular weight: Hsp100, Hsp90, Hsp70, Hsp60, and small Hsp (sHsp) [11]. Among them, Hsp70 is the most ubiquitous family. These family members are highly conserved in structure and contain two main functional domains: the nucleotide-binding domain (NBD) at the N-terminus, with a molecular weight of approximately 44 kDa, and the peptide-binding domain (PBD), with a molecular weight of approximately 25 kDa at the C-terminus. The flexibility of the variable C-terminal region is conducive to binding between the substrates and the SBD [9,12]. In plants, Hsp70s are divided into two subfamilies, DnaK and Hsp110/SSE [6]. Hsp70 is a ubiquitous molecular chaperone that helps proteins correctly fold in unfavorable environments, prevents protein aggregation or inactivation, and plays a central role in cellular protein quality control and degradation systems to promote cell tolerance to stress environments [13]. Currently, Hsp70 is not only responsive to a wide range of abiotic and biotic stressors in plants, including drought, high salinity, low-temperature stress, heavy metal toxicity, chemical exposure, and hormonal stimuli, but also plays a well-documented role in regulating HS responses, as evidenced by numerous studies [14]. Kozeko (2021) reported that AtHsp70-4 and AtHsp70-5 were highly induced under HS. Further knockout experiments revealed that AtHsp70-5 significantly contributed to the heat tolerance of plant seedlings [15]. In addition, after *Arabidopsis* Hsp70-14/15 gene silencing, the mortality caused by heat treatment sharply increases, and tolerance to the heat response weakens [16]. The overexpression of the pepper *CaHsp70-2* gene in *Arabidopsis* also induces the expression of related genes and endows transgenic *Arabidopsis* with increased heat tolerance [17]. Zhao et al. (2019) reported the same phenomenon after transferring Hsp70 from tree peony into *Arabidopsis thaliana* [18]. These results fully indicate that Hsp70 plays multiple regulatory roles in plant growth, development, and stress response, and is thus a vital resource for exploring plant stress response genes. To date, various biological functions of Hsp70 have been systematically identified and extensively studied in many plants, including radish (*Raphanus sativus*) [6]. These include apple (*Malus domestica*) [19], mango (*Mangifera indica*) [20], pineapple (*Ananas comosus*) [21], dates (*Ziziphus jujuba*) [22] and other horticultural plants.

The red-skinned and white-fleshed *Hylocereus undatus* (2*n* = 22) is a perennial climbing succulent herb belonging to the genus *Hylocereus* in the Cactaceae family. It is popular among growers and consumers due to its ease of cultivation, unique appearance, pleasant flavor, and richness in nutrients such as betalains, thus holding high commercial and medicinal value [23,24]. The optimal temperature for *H. undatus* growth ranges from 20 to 30 °C, and its unique Crassulacean acid metabolism (CAM) system enables it to grow and survive even at a high temperature of 40 °C, endowing it with strong heat tolerance [25]. Currently, research on *H. undatus* mainly focuses on the biosynthesis of betalains, while the mechanism underlying its tolerance and resistance to HS remains unclear. Moreover, the comprehensive identification and functional characterization of the Hsp70 gene family in *H. undatus* remain unelucidated [26]. To date, no investigations have been conducted on the role of the *HuHsp70* in the response to HS. With the availability of the chromosome-level genome assembly of *H. undatus* and the recent release of the *H. undatus* Genome and Multiomics Database (PGMD, http://pitayagenomic.com/ accessed on 21 March 2025), a reliable tool has been provided for identifying members of the Hsp70 gene family at the genome-wide level [26,27].

In this study, we identified the HuHsp70 gene family using bioinformatics approaches, analyzed the gene structure, subcellular localization, conserved motifs, evolutionary relationships, promoter cis-acting elements, and GO/KEGG enrichment of its members, and evaluated their expression profiles at different time points under HS by integrating transcriptome data. In addition, we proposed a specific regulatory model to illustrate how the *HuHsp70-11* gene clears excessive intracellular reactive oxygen species (ROS), thereby enhancing cell membrane permeability and further improving the heat resistance of *H. undatus* plants. This study provides a basis for understanding the structural and functional characteristics of *HuHsp70*, as well as candidate genes for further research on the molecular breeding of *H. undatus* for HS tolerance.

## 2. Results

### 2.1. HuHsp70 Identification and Physicochemical Properties of Gene Families

To obtain the *HuHsp70*-related gene ID and sequence, we used *Arabidopsis* Hsp70. The protein sequences of family members were used as a reference, and incomplete or nonexistent domains were removed through BLASTP alignment, HMMER searches, and NCBI-CCD domain analysis. Twenty-seven typical Hsp70 domains were ultimately obtained from the *H. undatus* genome. The gene family members are shown in Table S2. According to the chromosomal locations of these genes, the gene family members were renamed from top to bottom as *HuHsp70-1* to *HuHsp70-27*. We analyzed the physicochemical properties of the identified sequences, including molecular weight, theoretical pI, instability index, aliphatic index, grand average hydropathicity, and subcellular localization. The results showed (Table S2) that the number of HuHsp70 member proteins differed little, ranging from 607 aa (HuHsp70-16) to 702 aa (HuHsp70-6), and the molecular weights ranged from 67.10 kDa (HuHsp70-16) to 75.31 kDa (HuHsp70-19), with an average value of 71.80 kDa. The theoretical pI ranged from 5.09 (HuHsp70-3 and HuHsp70-8) to 8.58 (HuHsp70-22), with an average value of 5.84. Except for those of HuHsp70-13, HuHsp70-22, and HuHsp70-25, the isoelectric points of the other HuHsp70 proteins were less than 7. Therefore, we believe that the members of this family generally behave as acidic proteins. An instability index of less than 40 indicates that the gene will be translated into a stable protein. In this gene family, only three members (HuHsp70-1, HuHsp70-2, and HuHsp70-19) are unstable proteins. The aliphatic index was between 80.65 (HuHsp70-4) and 102.35 (HuHsp70-9), with an average of 86.35, both greater than 80. Except for HuHsp70-0 and 9, the average hydropathicity values of the other genes were all less than 0, ranging between −0.486 and 0.19, indicating that the HuHsp70 gene family members are all hydrophilic proteins. In addition, we also predicted the subcellular locations of these genes. The subcellular localization predictions via the Plant-mPLoc tool revealed (Table S2) that these proteins were located in multiple cellular structures, primarily the cytosol and cysks. The parameters of these HuHsp70 gene family members are basically similar to those of the genes in *A*. *thaliana* that have been proven to be related to HS response, indicating that they have the potential to participate in HS response.

### 2.2. Phylogenetic Analysis and Classification of the Hsp70 Gene Family in H. undatus

**Figure 1:**
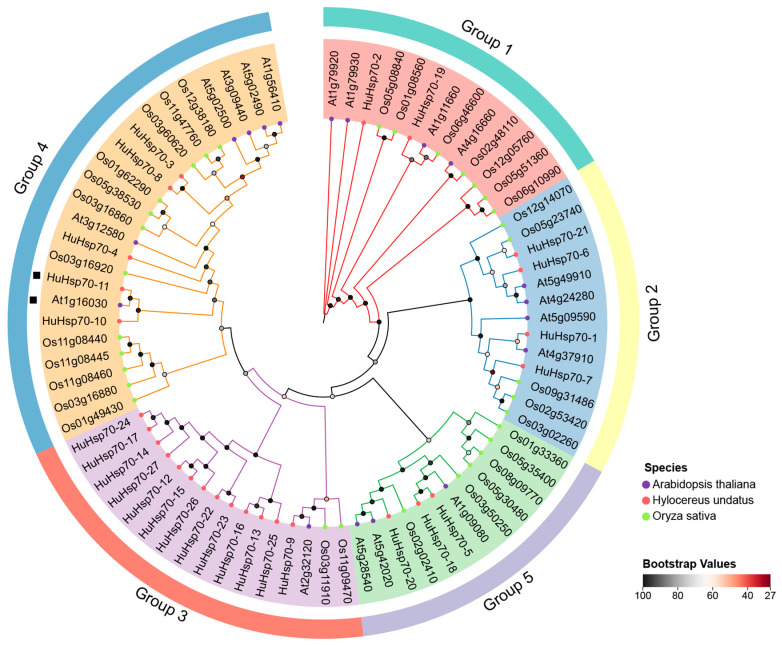
Phylogenetic relationships of Hsp70 proteins in *H. undatus*, Arabidopsis, and rice. The phylogenetic tree was constructed based on the HuHsp70, AtHsp70, and OsHsp70 sequences (the bootstrap value was 1000 repeats). The diverse colors represent the Group 1, Group 2, Group 3, Group 4, and Group 5 subfamilies, and the candidate genes are marked with black squares. Members of different species are represented by various symbols. The square symbol indicates At1g16030 and HuHsp70-11 which are clustered together, and At1g16030 is considered an important gene involved in heat shock response in *Arabidopsis thaliana*.

For an in-depth understanding of Hsp70 in terms of the evolutionary relationships between members, we used 27 HuHsp70, 18 AtHsp70, and 32 OsHsp70 protein sequences for alignment, and a phylogenetic tree was constructed. According to the topology of the phylogenetic tree (Figure 1), we divided the Hsp70 sequences from different species into five categories: Group 1, Group 2, Group 3, Group 4, and Group 5. Among them, Group 4 contained the most genes, with 23 members, including five HuHsp70s, six AtHsp70s, and 12 OsHsp70s. However, Group 3 had the highest number of HuHsp70s, with 13 members accounting for 48% of HuHsp70. The HuHsp70 proteins cluster with Arabidopsis Hsp70 more frequently in the smallest distance (three homologous gene pairs) than with rice (one homologous gene pair), suggesting closer evolutionary relationships between H. undatus and dicotyledonous plant Hsp70 genes. These results suggest that, compared with that of rice (monocot), the phylogenetic pattern of *H. undatus* is more similar to that of dicots, especially *Arabidopsis*.

### 2.3. Gene Structure and Conserved Motifs of HuHsp70s

**Figure 2:**
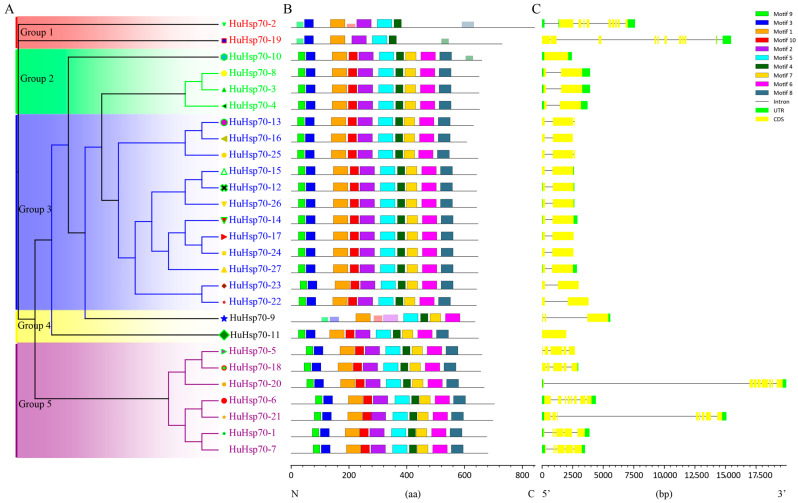
Phylogenetic tree, conserved motifs, and gene structure of HuHsps. (**A**) MEGA 7.0, which is based on the HuHsp70 phylogenetic tree constructed from protein sequences, was used, and the names of the groups were annotated accordingly. (**B**) Distribution of conserved motifs in HuHsp70 proteins. The icons in different colors represent different motifs, from Motif 1 to Motif 10. Table S3 provides detailed sequence information for each motif. (**C**) Conserved domain and gene structure of the HuHsp70 protein. The figure was visualized via CFVisual software (V 2.1.5).

To investigate the structural and functional characteristics of the gene family member sequences of *HuHsp70*, we analyzed the gene structure and conserved motifs of all the Hsp70 genes in *H. undatus*. First, we used the 27 identified *HuHsp70* sequences to construct a phylogenetic tree to analyze the evolutionary relationships among the different members of the Hsp70 gene family (Figure 2A). We divided these genes into five subfamilies according to their topological structure. Group 3 contained the most genes, Group 1 and Group 4 the least, with each subfamily having only two genes, which was far less than the number of genes in the other three subfamilies. Second, this study also analyzed the motif composition of *HuHsp70* via the MEME online tool. The results (Figure 2B) revealed that the 10 predicted motifs were highly conserved in this gene family; 88.9% of the sequences contained the above motifs, and they were very similar in quantity and distribution pattern. However, the positions of the same motif in different sequences differ, and there is a gap between them, which may affect protein function. In general, the conserved motif structures of this family are highly conserved, which also provides the basis for them to perform the same function. Notably, the two genes in Group 1 differed in the composition and quantity of conserved motifs, indicating that the *HuHsp70-2* and *HuHsp70-19* genes in this subfamily may be involved in specific functions, which may be vital factors in some environments. This is caused by the evolution of the body under stress, and we will conduct an in-depth analysis of these two genes in future studies. Differences in gene structure may lead to changes in the distribution of conserved HuHsp70 motifs. Therefore, we analyzed the exon/intron structure of these 27 *HuHsp70* genes based on their genome annotation files. The results revealed that (Figure 2C) the gene structure of most *HuHsp70s* is highly conserved. Each gene contained at least one CDS fragment and a maximum of 10, and the number of UTRs was no more than three. Based on the results of the phylogenetic tree, we considered that the structure of the genes in the same clade was more similar and that the number and distribution of exons and introns in different clades were significantly different. Group 4 of the phylogenetic tree (Figure 1) comprises five *HuHsp70* genes (*HuHsp70-3*, *HuHsp70-4*, *HuHsp70-8*, *HuHsp70-10*, and *HuHsp70-11*). While some genes appear to belong to different branches in terms of gene structure and conserved motif clustering, their bootstrap values in the phylogenetic tree confirm they belong to the same subfamily. Differences in gene structure and conserved motifs may result from functional diversification during evolution (e.g., adaptation to distinct subcellular localizations or stress response scenarios). This phenomenon of “evolutionary clustering with structural divergence” is common among gene families and suggests functional diversity. In general, the composition, number, and length of the *HuHsp70* genes were roughly similar, indicating that the structure of the genes of this family was strongly conserved. The structure of these HuHsp70 gene family members is similar to that of the related genes in *A*. *thaliana*, suggesting that they may have the potential to be involved in HS response.

### 2.4. Homeopathic Element Analysis of the Promoter Sequence of the Hsp70 Gene in H. undatus

**Figure 3:**
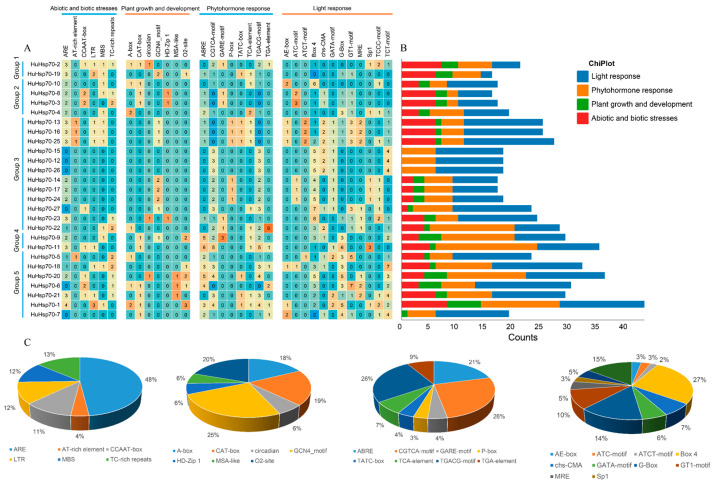
Prediction of cis-acting elements in promoters. (**A**) The number of cis-acting elements contained in each *HuHsp70* promoter is divided into four types. (**B**) *HuHsp70* response elements in promoters, including the types and quantities of elements. (**C**) Pie graph showing the proportion of each promoter element among the four types of response elements.

To understand *HuHsp70* and investigate the function and regulatory role of the gene in biological processes, we used the Plant CARE website to predict the cis-acting elements in the upstream 2000 bp region of the gene. The analysis results revealed that (Figure 3), in addition to the TATA box, CAAT box, and some elements with unknown functions and conserved sequences, we predicted 640 cis-acting elements and classified them. These include abiotic and biotic stresses, plant growth and development, and phytohormone and light responses, with the proportion of the number of components in each category accounting for 15.9%, 8%, 29.2%, and 46.9%, respectively. We identified many ARE regulatory elements associated with abiotic and biotic stresses, accounting for 48%; their main functions were related to anaerobic induction and were predicted in multiple *HuHsp70s*. In addition, 12 LTR elements directly related to the temperature response were detected, and three genes related to these elements were mainly concentrated in Group 3. The plant growth and development response elements included the A-box, CAT-box, circadian, GCN4_motif, HD-Zip 1, MSA-like, and O2-site. O2-site elements regulated by protein metabolism accounted for a relatively high proportion. The phytohormone response elements include methyl jasmonate (CGTCA motif and TGACG motif), salicylic acid action elements (TCA elements), gibberellin action elements (GARE motifs, P boxes, and TATC boxes), abscisic acid response elements (ABREs), and auxin action elements (AuxRR cores and TGA elements). Notably, ABREs (21%) are closely related to the biosynthesis of abscisic acid, a vital substance for plants to resist high-temperature stress. We detected this element in a large number of *HuHsp70* promoters. In addition, the promoter of *H. undatus* Hsp70 also contains many light-responsive elements, e.g., Box4 (27%), the G-box (14%), and the TCT motif (15%). These light response elements include *HuHsp70* genes, which can participate in various light-related responses, such as photosynthesis. As shown in Figure 3, several HuHsp70 family members, e.g., HuHsp70-7, HuHsp70-12,HuHsp70-15, and HuHsp70-26, lack typical stress-responsive cis-elements, which appears anomalous for heat shock proteins known for their stress-inducible nature. This observation may reflect functional divergence within the gene family, where certain members have evolved tissue-specific or constitutive expression patterns independent of canonical stress signaling pathways. Alternatively, these genes might rely on non-canonical regulatory elements or epigenetic modifications to mediate stress responses, warranting further experimental validation of their transcriptional regulation mechanisms.

In summary, we believe that the 640 predicted cis-acting elements can regulate the transcription level of genes in plants under stress and improve the synthesis and metabolism of resistant substances, enabling plants to adapt better under unfavorable conditions. The above analysis of promoter cis-acting elements revealed that the expression of Hsp70 gene family members may increase the adaptability of *H. undatus* seedlings under various stress conditions, such as high-temperature stress. Among these genes, the number of ARE elements reached 49, which can play a balancing role when reactive oxygen species are produced in large quantities. In addition, elements such as LTRs, AT-rich repeats, and TC-rich repeats, which can maintain protein binding and normal function after being subjected to temperature stress, were also retrieved in some of the genes.

### 2.5. Mapping and Collinearity Analysis of the HuHsp70 Gene

To understand the chromosome distribution and genome-wide density of the *H. undatus* Hsp70 gene, we performed chromosome location analysis via TBtools software (II v2.136). The results (Figure S1) revealed 27 *HuHsp70* genes that were randomly distributed on nine chromosomes (Chr1, Chr2, Chr3, Chr5, Chr6, Chr7, Chr8, Chr9, and Chr11). In addition to Chr4 and Chr10, all the chromosomal scaffolds contained at least one *HuHsp70* gene, but the overall distribution was uneven. These genes are distributed on the Chr7 chromosome. Notably, Chr7—where the greatest number of *HuHsp70* genes (8) are located—is of particular interest, as Hsp70 family genes are widely reported to be involved in plant response to HS, suggesting these clustered *HuHsp70* genes may play a key role in *H. undatus* heat tolerance. The number of genes was the greatest, reaching 8, while there were 1 to 6 *HuHsp70* genes on other chromosomes, and the number distribution was uneven. Moreover, we also found that Chr7 and Chr11, which have the greatest number of genes, are mostly concentrated in a certain position. Chromosome length has no direct relationship with the number of genes. Therefore, we speculate that this phenomenon is caused by gene duplication, which will be further explored in our study.

**Figure 4:**
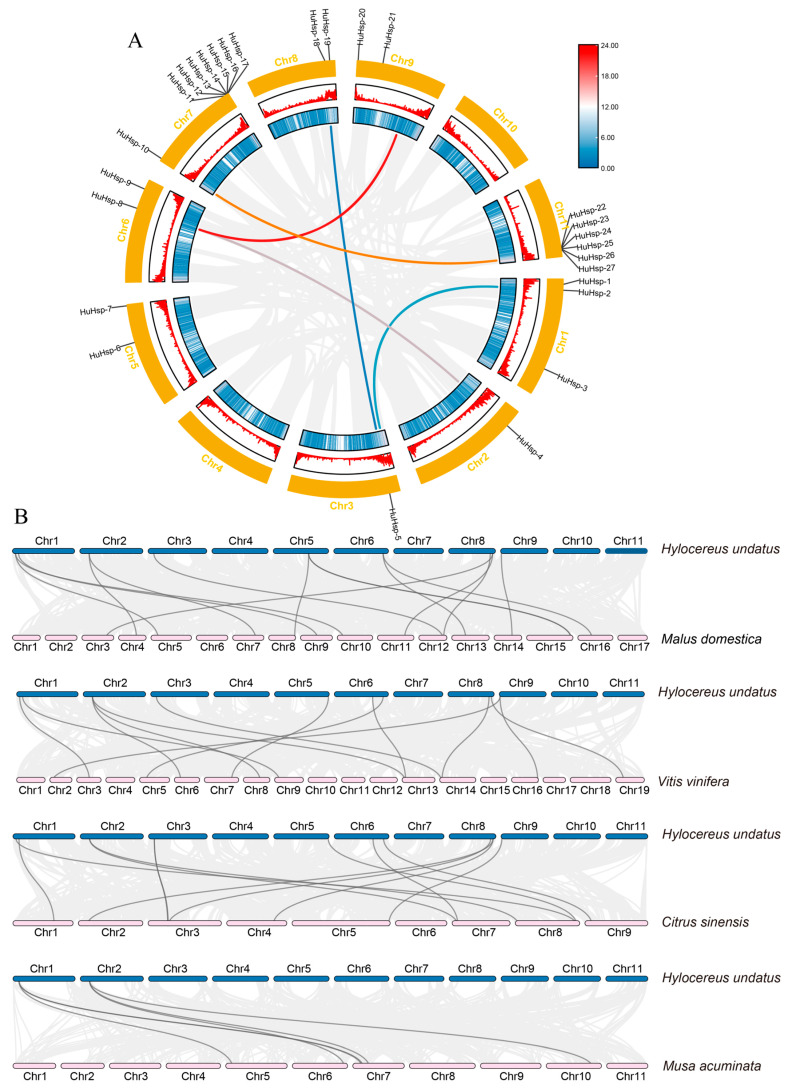
*HuHsp70* collinearity analysis of gene family members. (**A**) Intraspecific collinear analysis of 27 *HuHsp70* genes. The gray arc area in the background represents all the collinear regions in the *H. undatus* genome. Genes connected by arcs and with the same text color have a collinear relationship, that is, homologous gene pairs. The chromosome name and number are marked outside of the chromosome bracket. (**B**) Interspecific collinearity analysis of genes in the *H. undatus*, apple, grape, sweet orange, and banana genomes of Hsp70. The red line between the two genomes indicates the Hsp70 gene pairs with collinear relationships. The gray arc area represents all the collinear regions in the *H. undatus* genome and the target genome. The chromosome number is annotated at the top or bottom of the corresponding backbone.

Gene duplication events are critical to the evolution of family members and play a vital role in the evolution of organisms. Therefore, we used the MCScanX program to analyze 27 *HuHsp70* gene duplication events. Collinearity analysis of the *H. undatus* genome revealed that (Figure 4A), in the Hsp70 gene family, five gene duplication events occurred, i.e., *HuHsp70-10/HuHsp70-27*, *HuHsp70-8/HuHsp70-6*, *HuHsp70-5/HuHsp70-18*, *HuHsp70-4/HuHsp70-8*, and *HuHsp70-2/HuHsp70-1*. The results of the phylogenetic tree of the HuHsp70 family (Figure 2A) revealed that the gene duplication events all occurred in the same evolutionary clade and were closely related. This finding indicates that the replicated gene pairs are highly similar in structure and function but evolve in different directions. Given that *Hsp70* genes are core regulators of the plant HS response, the conservation of these duplicated *HuHsp70* pairs under purifying selection may reflect their essential role in maintaining *H. undatus* adaptation to heat environments. To further explore the relationships between the replication and differentiation of HuHsp70 gene family members and selection pressure, we calculated Ka (nonsynonymous mutation rate), Ks (synonymous mutation rate), and the Ka/Ks (ratio of the nonsynonymous mutation rate to the synonymous mutation rate) to estimate the selection pressure for the effects of Hsp70 on gene duplication and differentiation. The results (Table S4) revealed that the Ka/Ks values of these replicated gene pairs were all less than 1, indicating that these genes were affected mainly by purifying selection pressure. In addition, the Ks value ranged from 0.93~3.54, and it can be speculated that the differentiation time was 71.17 million years ago (Mya)~272.19 Mya (Table S4). In addition, we selected four common fruits, namely, apples, grapes, sweet oranges, and bananas, to analyze the collinear relationship between them and *H. undatus* concerning Hsp70. The results (Figure 4B) revealed 15, 13, 13, and six homologous genes, respectively. We found that bananas presented the fewest number of homologous genes, apples presented the greatest number, and grape and sweet orange presented the same number of genes, both 13.

### 2.6. H. undatus Hsp70 Gene Expression Profiles Under HS

To further understand *HuHsp70* and investigate the underlying molecular mechanisms at the expression level, we performed transcriptomic analysis. The experimental material was tissue-cultured seedlings of Guanhua white *H. undatus*. The samples were placed in a high-temperature environment at 40 °C for 24 h and 48 h, respectively. Then, RNA was extracted from the stem segments of the seedlings, which were constructed after passing quality control and subsequently used in the future. Computer sequencing was performed on the next-generation high-throughput sequencing platform to obtain the transcriptomic data of *H. undatus* after different periods of HS. In the first step, the off-machine data were filtered to obtain clean data, which were aligned with the specified reference genome (*H. undatus* v1) to seek mapped data. In the second step, based on the alignment results and the position information of the gene on the reference genome, the number of reads for each gene was counted. Finally, to ensure that the number of fragments reflected the gene expression level, we normalized the number of mapped reads and the transcript length in the samples. In this study, we used the FPKM value as an indicator to measure gene expression levels. In addition, we obtained the expression level of the Hsp70 gene family in *H. undatus* seedlings during HS from the RNA-seq data via the featureCounts software (V 2.1.1) (Table S5).

**Figure 5:**
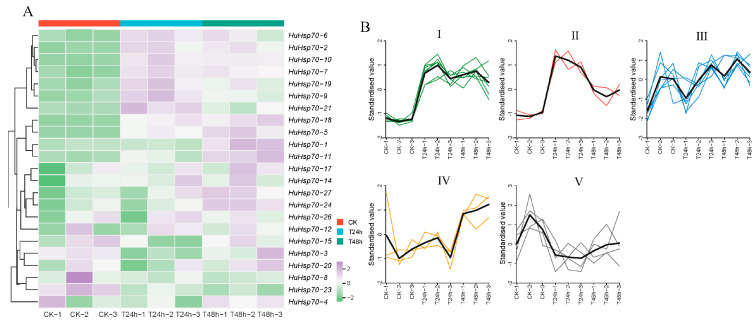
Expression profile and K-means cluster analysis of Hsp70 in *H. undatus* seedlings under HS stress. (**A**) Heatmap showing *HuHsp70*. For the clustering and distribution of quantitative data under HS stress, each data point was analyzed via the FPKM value. The z-score was normalized through processing. The color scale represents the normalized data. (**B**) Expression of *HuHsp70* in *H. undatus* seedlings: K-means clustering and expression trend analysis. The horizontal axis represents the samples in different periods, and the vertical axis represents the expression levels after the z-score transformation. Different colors represent different expression trends of *HuHsp70*. I–V are groups of different gene expression trends.

Among differentially expressed genes (DEGs), the majority (9 DEGs at 24 h, 11 DEGs at 48 h) exhibited an upward trend (Figure 5A). Furthermore, the expression levels of certain genes (e.g., *HuHsp70-11*, *HuHsp70-18*) exhibited significantly elevated expression levels with prolonged HS duration. The HuHsp70 gene family exhibits diverse responses to HS, with most DEGs upregulated and a few genes showing no significant change or weak downregulation. In addition, we performed k-means clustering and expression trend analysis on these 23 *HuHsp70s* and divided them into five groups according to trends in their expression levels (Figure 5B). We named these Groups I to V, which contained seven, two, seven, three, and four *HuHsp70* genes, respectively. In addition, according to the identification conditions of the DEGs, all the DEGs identified in this study were ultimately obtained through pairwise alignment between different sample groups (Figure S2). At 24 h, we identified eight DEGs, which increased to 9 at 48 h. These findings indicate that as HS continues, an increasing number of Hsp70 genes play roles. In addition, we also found that under HS, the expression levels of eight *Hsp70* genes (*HuHsp 70-1*, *HuHsp70-5*, *HuHsp70-11*, *HuHsp70-17*, *HuHsp70-18*, *HuHsp70-24*, *HuHsp70-26*, and *HuHsp70-27*) increased with increasing duration of stress. Notably, *HuHsp70-1* and *HuHsp70-11* appeared as DEGs at 24 h and 48 h, and their expression levels continued to rise under HS. These two genes were both ranked in group IV in the K-means cluster analysis, so we also considered that the expression of this group of genes could respond positively to HS. It is worth noting that the *HuHsp70-1* promoter region contains 3 ABRE elements and 3 LTR elements, and the *HuHsp70-11* promoter region contains 6 ABRE elements and 1 LTR element, both of which are significantly higher than other non-DEG genes. These findings indicate that the *HuHsp70-1* and *HuHsp70-11* genes may play key roles in the regulatory network of *H. undatus* seedlings in response to HS.

### 2.7. GO Functional Annotation and KEGG Pathway Enrichment Analysis

**Figure 6:**
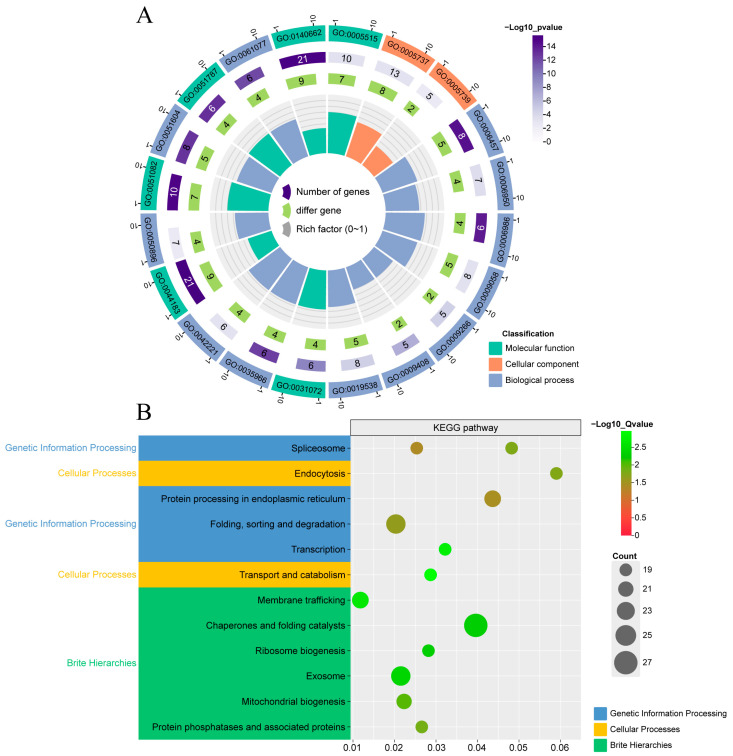
(**A**) displays the results of the GO enrichment analysis in a circular layout. From the innermost to the outermost layer: the first circle indicates the ratio of genes enriched in the same GO term to the total number of background genes. The second circle represents the combined count of upregulated and downregulated genes enriched in the GO term. The third circle shows the total number of genes enriched in the corresponding GO term. The fourth circle labels the GO identifier, and distinct colors represent GO categories. The numbers outside the circle indicate the scale of enriched gene counts. Biological processes, cellular components, and molecular functions are represented in different colors. (**B**) Scatter plot of the KEGG pathway enrichment results, with each point representing a KEGG pathway.

To further elucidate the biological function of the *HuHsp70* gene, the present study used the EggNOG-MAPPER database based on the above transcriptome data. Functional annotation and pathway enrichment analysis were subsequently performed via the online tool ChiPlot. The results of the GO functional annotation (Figure 6A, Table S6) revealed that 22 *HuHsp70* genes (81.5%) were successfully annotated in the database. The results were assigned to three categories: molecular function (molecular function, MF), biological process (biological process, BP), and cellular localization (cellular component, CC). This study revealed 20 GO terms, each enriched with more than five genes; the GO terms with the most enrichment reached 21 genes, and all were classified as molecular functions. Among the top 20 GO terms, 12 were associated with biological processes, accounting for more than 60% of the total proportion. Therefore, we believe that Hsp70 is involved in many vital functions and biological processes in plants. Biological processes mainly include responses to stimuli and pressure, protein folding, and metabolism. Notably, GO:0009408 and GO:0009266 are related to the response processes to temperature and HS, so we believe that some members of this family can actively respond to plant resistance to HS. Among the enriched CC terms, the only enriched GO terms were mitochondria (GO:0005739) and cytoplasm (GO:0005737). In addition, molecular function is related mainly to protein-folding partners and the binding of folded proteins. Notably, GO:0031072, which has terms related to heat shock protein binding and is closely related to the subject (Hsp70) in this study, was used. Moreover, *HuHsp70-11* was also annotated in these GO terms. KEGG pathway enrichment analysis revealed (Figure 6B) that all 27 genes of the HuHsp70 gene family were assigned to 12 KEGG pathways. These pathways include five main categories, namely, genetic information processing, cellular processes, and brite hierarchies. Pathways such as ribosome biogenesis, exosome, mitochondrial biogenesis, protein phosphatases, and associated proteins were significantly enriched, and they were related to protein processing, folding, sorting, and degradation. These results indicate that members of the HuHsp70 gene family play vital roles in the process of HS in *H. undatus* seedlings—*HuHsp70*-11—whose KEGG pathway involves protein processing in the endoplasmic reticulum, etc.

### 2.8. Quantitative Real-Time PCR Validation

**Figure 7:**
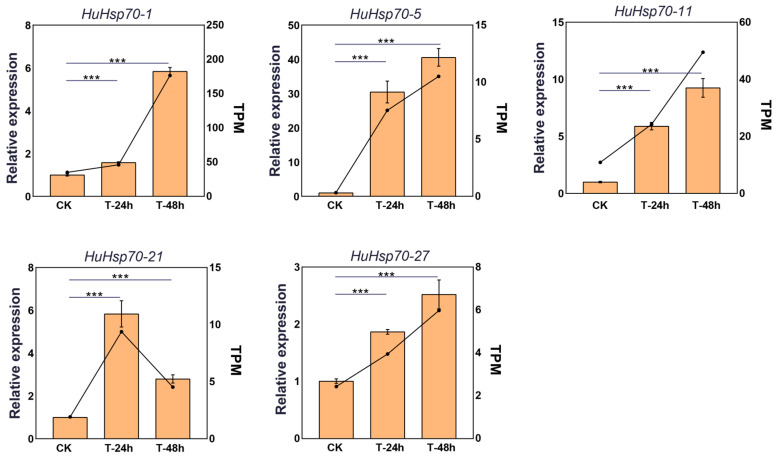
Five *HuHsp70* qRT-PCR validations of the *Actin* gene (HU07G00022) were used as internal reference genes for the relative expression level. All the data are averages of three biological replicates, and the error bars represent the standard deviation of three biological replicates. *** represents a significant difference among treatments at different time points (*p* < 0.001).

In this study, we analyzed five HuHsp70 gene family members via qRT-PCR. The results (Figure 7) revealed that the expression trends of the genes detected via qRT-PCR were consistent with those obtained via RNA-seq analysis. These findings indicate that the RNA-seq data in this study are reliable and can effectively reflect the gene expression levels of *H. undatus* seedlings during HS.

## 3. Discussion

*H. undatus* is one of the most widely planted tropical fruits worldwide and has high economic value [28]. In recent years, owing to the intensification of climate change and the frequent occurrence of extreme weather, the development of the *H. undatus* industry has been severely affected. Therefore, at present, it is crucial to breed new *H. undatus* varieties that are resistant to high-temperature environments, mine HS genes, and study the tolerance mechanisms of HS. HS can cause severe development and fertilization disorders in the vegetative and reproductive stages of *H. undatus*, thereby reducing fruit yield and quality [25]. Hsp70 is a conserved and ubiquitously expressed family among the heat shock protein families. It usually functions as a molecular chaperone and participates in the folding and transport of nascent proteins, the refolding of denatured proteins, and the degradation of denatured proteins [13,29,30]. Although the transcriptional regulatory network of Hsp70 gene family members under HS has been characterized in some fruit and vegetable crops, their systematic identification and functional characterization in *H. undatus* have not been explored [11,31]. Therefore, this study comprehensively identified and characterized *HuHsp70*. The functions of gene family members were also investigated. The potential role of the *HuHsp70-11* gene in the HS regulatory network was also explored.

In this study, 27 *Hsp70* genes in the *H. undatus* genome were identified via bioinformatics methods. We also performed correlation analysis on their physicochemical properties, and the average molecular weight of all the genes was 71.80 kDa. The Hsp70 genes were identified at 67.10 kDa (HuHsp70-16) ~ 75.31 kDa (HuHsp70-19), which meets the definition of Hsp70 gene family members and supports the correction of our identification results [32]. The number of *Hsp70* genes in *Raphanus sativus* (25) [6], *Arabidopsis* (18), and *Capsicum annuum* (21) [17] was relatively greater but lower than that in *Glycine max* (61) [33], *Brassica napus* (47) [34], and rice (32), indicating that the number of Hsp70 genes in different species is different and has no correlation with genome size. We subsequently conducted multiple sequence alignments with related genes in Arabidopsis and rice to construct a phylogenetic tree (Figure 1). With respect to the topological structure of genes encoding Hsp70, we considered these genes to encode Hsp70. The genes were classified into Group 1, Group 2, Group 3, Group 4, and Group 5, which was consistent with previous studies [33]. Notably, the number of *HuHsp70* genes in Group 3 reached 13, almost half of the number of *HuHsp70* genes in the entire gene family. Therefore, it is believed that this subfamily may play a key role in *H. undatus* resistance to HS; it may collaborate in heat resistance, with different members specializing in ROS scavenging, maintaining membrane stability, and other functions. In addition, we also found that in specific evolutionary clades, *H. undatus* Hsp70 clustered with more members from *A*. *thaliana*, which was consistent with the results of He et al. (2023) [6]. These findings also indicate that *H. undatus* and *Arabidopsis* have a close evolutionary relationship. We can also speculate on the potential function of the corresponding genes in *H. undatus* through the function of the AtHsp70 gene. Therefore, if two genes are in the same subfamily, we can speculate on the function of unknown genes from genes with known functions [35]. According to the phylogenetic relationship analysis of the structure and conserved motifs of 27 *H. undatus* Hsp70 genes, genes from the same subfamily were found to have similar gene structures and motif compositions, which further validated the taxonomy and phylogeny of the HuHsp70 family. In this study, we predicted and detected ten motifs. A total of 88.9% of the genes contained the above ten motifs, and only three had partial motif deletions. Therefore, genes in this family were highly conserved during the evolutionary process. Gene structure and motif composition do not undergo prime changes [36]. We also found that Group 3 had the highest number of genes, far more than the other subgroups did; therefore, we considered that genes in this subgroup might perform the same function. Group 3 contains 13 *HuHsp70* genes. The gene structure and motif composition of this subfamily are highly conserved. Three genes contain heat-responsive LTR elements, and four genes contain response-related elements ABREs. It is speculated that Group 3 is the core subfamily of *H. undatus* that responds to HS. From the perspective of gene structure, the members of the HuHsp70 gene family are also highly conserved, with the compositions of the CDS and UTR being very similar, which further supports the conclusion that the Hsp70 gene is highly conserved [6].

Cis-acting elements are regulatory factors that play crucial roles in gene transcription [35]. In this study, in addition to the TATA box, CAAT box, and several elements with unknown functions, the cis-acting elements in the promoter region of the *HuHsp70* gene included mainly abiotic and biotic stresses (102) and plant growth and development (51). For the phytohormone response (187) and light response (300) genes, 640 cis-acting elements were predicted. Most Hsp70 promoters are forecasted to contain the ABA response element ABRE, and ABREs are abundant. These findings indicate that under HS, *HuHsp70* can improve plant resistance to adverse conditions by regulating hormone levels and substance synthesis, thereby responding to adverse environments. In addition, LTR, AT-rich, and TC-rich repeat elements, which can maintain the regular binding and function of proteins after being subjected to temperature stress, are abundant in these genes. By constructing a distribution map of the *HuHsp70* gene on chromosomes, we found that, with the exception of Chr4 and Chr10, the other chromosome scaffolds all contained at least one Hsp70 gene, and the distribution was uneven. To clarify the evolutionary relationships of these genes, we performed intraspecific collinearity analysis and detected Hsp70 gene duplication events. Five gene duplication events occurred, that is, gene duplication of Hsp70. The number and evolution of genes in this family play important roles. The gene duplication event generated a new Hsp70 gene for the species, which provided a new direction and impetus for the evolution of the HuHsp70 gene family and helped *H. undatus* plants face HS [37]. In addition, based on the gene sequences of the gene duplication events, we calculated Ka, Ks, and Ka/Ks for the replicated gene pairs to assess the selection pressure on the Hsp70 gene during replication and differentiation. The Ka/Ks values of these replicated gene pairs were all less than 1, indicating that these genes are affected mainly by purifying selection pressure. In addition, the Ks values ranged from 0.93 to 3.54, suggesting that the differentiation time was between 71.17 Mya and 272.19 Mya, indicating that the differentiation event of the HuHsp70 gene family occurred after the differentiation of dicots and monocots. This finding further illustrates that gene duplication events are vital for new gene generation and promote the functional differentiation of the HuHsp70 gene family, which is consistent with the results of Ma et al. (2021) [38]. In addition, this study further compared the collinear relationships between *H. undatus* and four common fruit crops. The collinearity between *H. undatus* and apple was greater than that between grape, sweet orange, and banana. It indicated that the Hsp70 gene of *H. undatus* is highly evolutionarily conserved.

When plants are subjected to adverse stress, cells activate gene expression programs and regulate metabolite accumulation, maintaining normal intracellular activities and adapting to new environmental conditions [39]. In this study, we used transcriptome data and bioinformatics to analyze the trend of Hsp70 gene expression in *H. undatus* seedlings under HS, and we also verified the accuracy of the transcriptome data via qRT-PCR. In the present study, 85.2% of the *HuHsp70* genes were expressed at 24 h and 48 h, whereas the expression levels of the other four genes did not reach 0.1. According to the DEG screening results, eight DEGs were detected at 24 h, and nine DEGs were detected at 48 h. Moreover, the expression levels of HuHsp70 -11 and other genes continued to increase. It illustrated that as HS continued, *H. undatus* seedlings gradually activated more Hsp70 genes to enhance stress resistance, and the Hsp70 gene can play a vital regulatory role under HS. The number of DEGs and the fold change are constantly increasing. This may also be due to the continuous prolongation of the HS time. To survive, *H. undatus* seedlings have increasingly activated the *HuHsp70* gene or increased the expression level of this gene in response to the continuous prolongation of HS. Among these *HuHsp70* genes, the expression levels of *HuHsp70-1* and *HuHsp70-11* continued to increase over time, with *HuHsp70-11* expressed as a DEG at 24 h and 48 h. In contrast, *HuHsp70-1* also manifested as a DEG at 48 h, and its expression level also increased at 24 h, but this condition did not meet the conditions for DEGs. However, the expression level of *HuHsp70-11* increased significantly. Therefore, *HuHsp70-11* is a key candidate gene involved in the response of *H. undatus* seedlings to HS. In addition, we performed GO functional annotation and KEGG pathway enrichment analysis on these Hsp70 genes. The biological processes enriched in the GO analysis included responses to stimuli and pressure, protein folding, and metabolism, among which *HuHsp70-11* was significantly enriched in “replies related to temperature and heat stress”. The cellular components included mainly mitochondria and the cytoplasm, which were essentially consistent with the predictions of subcellular localization. In terms of molecular function, it is relevant mainly to protein folding partners and the binding of folded proteins. The enriched KEGG pathways were primarily related to the processing, folding, sorting, and degradation of proteins, and *HuHsp70-11* was assigned to multiple KEGG pathways. These results are essentially consistent with the function of Hsp70 in model plants. These results not only validate the accuracy of HuHsp70 gene family member identification but also indicate that *HuHsp70-11* plays a crucial role in regulating HS responses in *H*. *undatus* seedlings. Notably, this gene emerges as a key candidate for mediating high-temperature tolerance in *H*. *undatus* fruits. We will perform functional validation, such as transgenic overexpression and virus-induced gene silencing (VIGS), in follow-up studies.

**Figure 8:**
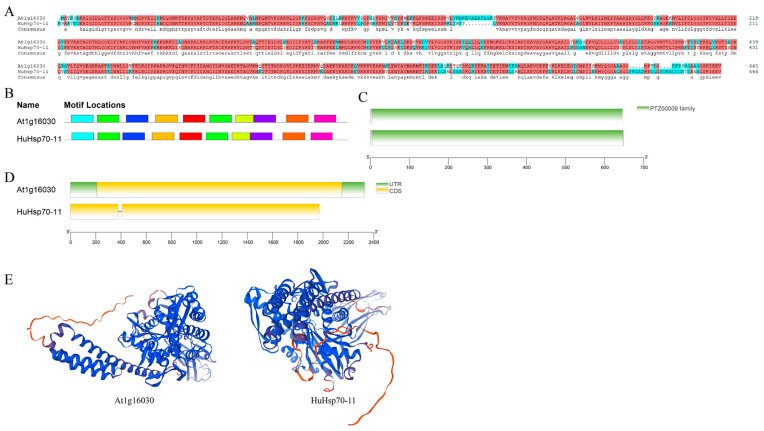
Comparative analysis of At1g16030 and HuHsp70-11. (**A**) Sequence comparison plot. (**B**) Conserved motif map. (**C**) Conserved structural domain map. (**D**) Gene structure diagram. (**E**) Tertiary structure diagram.

Hsp70 gene family members in *A. thaliana* are essential for plant development and heat tolerance in germinating seeds [40]. We characterized and converted these findings to IDs using TAIR (https://www.arabidopsis.org/, accessed on 22 March 2025) and found that they were almost the same as At1g16030 in this study. Notably, At1g16030 and *HuHsp70-11* are on the same evolutionary branch in the evolutionary tree and are closely related (see Figure 1); therefore, we believe that *HuHsp70-11* may also have similar regulatory effects on HS and are essential for plant development and for thermotolerance [40]. Sequences can be considered to be generally highly functionally conserved when their similarity is greater than 60% [41]. We analyzed these genes via Clustal Omega (https://www.ebi.ac.uk/jdispatcher/msa/clustalo, accessed on 9 January 2026) for multiple sequence comparisons and sequence similarity calculations and reported that the sequence similarity of the two genes was as high as 89.8% (Figure 8A). If HuHsp70-11 and At1g16030 are functionally conserved, they may exert similar effects, such as ROS scavenging and protein folding, by regulating known heat tolerance pathways in Arabidopsis. On this basis, we therefore performed a comparative analysis of these two genes, including the 3D structure, gene structure, conserved motifs, and conserved structural domains. The results (Figure 8B–D) revealed that both of these genes contain the structural domain of Hsp70 (PF00012) and that the composition and location of the conserved motifs are very similar. In terms of gene structure, At1g16030 has only two more UTR regions than *HuHsp70-11*, and the coding region has one fewer intron with a length of 29 bp; however, the overall length of the CDS differs by only two bp. For the physicochemical properties (Table S7), the magnitude of the values of each index was similar, and there was only a slight difference. Combined with the gene structure analysis (Figure 8D), we hypothesize that this is due to the presence of small segments of the UTR at the ends of the At1g16030 gene and the presence of a segment of the intronic region in *HuHsp70-11*, which leads to minor differences between the two genes in the above analysis. In addition, the subcellular localizations are all shown to be in cyto, which implies that the sites of action are also the same, further providing evidence for functional similarity [35]. We also submitted both genes to the TAIR and PGMD databases for KEGG functional enrichment searches. The results revealed that the GO enrichment of both was concentrated at GO:0009408 and GO:0009266, and the KEGG was enriched in the same pathway (protein processing in the endoplasmic reticulum), which is related to protein processing, folding, sorting, and degradation. In addition, the three-dimensional structures of the two genes were predicted and found to be very similar (Figure 8E). These findings further confirm that *HuHsp70-11* is functionally equivalent to the At1g16030 gene in Arabidopsis, which is vital for alleviating HS in *H. undatus*.

**Figure 9:**
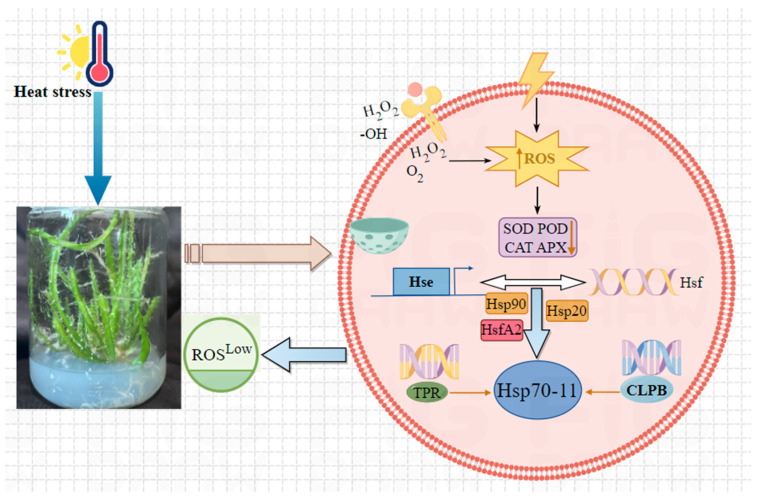
A potential model by which *HuHsp70-11* positively regulates the ROS content to promote cell membrane permeability. APX, Ascorbate Peroxidase; CAT, Catalase; CLPB, Caseinolytic Peptidase B homolog; POD, Peroxidase; SOD, Superoxide Dismutase; TPR, Tetratricopeptide Repeat.

Based on the cumulative findings from the above studies, we propose a specific regulatory model to delineate how the *HuHsp70-11* gene mediates the scavenging of excess intracellular reactive oxygen species (ROS). This process enhances cell membrane stability, thereby conferring HS tolerance in *H*. *undatus* (Figure 9). Under high-temperature stress in plants, the overproduction of reactive oxygen species (ROS) is one of the key factors that causes cell damage. When *H. undatus* plants experience high-temperature stress, a series of adverse reactions, such as photosynthesis, occur, and the most significant change is the overproduction of ROS. ROS include mainly superoxide anions (O^2−^), hydrogen peroxide (H_2_O_2_), hydroxyl radicals (-OH), etc., and the generation of ROS is primarily from the generation of ROS in photoreactions and ROS leakage in the mitochondrial electron transport chain (ETC). These overproduced ROS are among the key factors leading to cell damage. To resist this stress, plants gradually remove ROS by initiating signal transduction, activating gene expression, synthesizing antioxidant enzymes and non-enzyme substances, and promoting their synergy. At this time, the antioxidant enzyme system is activated to scavenge free radicals such as SOD, POD, CAT, and APX and catalyze the degradation of ROS into H_2_O and O_2_. However, this process is regulated by multiple signals. HS directly induces conformational changes in Hsf in plant cells and binds to and oligomerizes with Hse, causing it to migrate from the cytoplasm to the nucleus. Upon recruitment of coactivators such as Hsp20, Hsp90, and HsfA2, Hsp70 undergoes a conformational shift that alleviates its auto-repression, induces self-phosphorylation, and triggers robust transcriptional activation of its target genes. As a molecular chaperone, Hsp70-11 continues to bind to Hsf and is released to assist in the refolding of misfolded proteins. It also degrades irreparable proteins through the ubiquitin-proteasome system, thereby increasing the fluidity of cell membranes. This eventually reduced the ROS content to the normal level, which highly improved the antioxidant ability of *H. undatus* and resistance to HS. Notably, this study is based solely on tissue-cultured seedlings in vitro and has not verified gene function under naturally high temperatures in the field. The molecular mechanism of *HuHsp70-11* also requires further validation through transgenic experiments. In the future, we plan to validate the function of *HuHsp70-11* via virus-induced gene silencing (VIGS) or overexpression transgenics.

## 4. Materials and Methods

### 4.1. Plant Material and Treatments

The variety used in the experiment was ‘Guanhua’ white *H. undatus*, which is a clonal seedling obtained through a plant tissue culture technique with identical genetic background information. It was placed in the tissue culture laboratory of Southwest Forestry University. Seedlings with basically consistent growth and a height of 8 cm were selected, placed in a GXZ-2808 climate light incubator (Ningbo, China), and treated at 40 °C for 24 h (T-24) or 48 h (T-48), with three biological replicates per group. Seedlings grown at room temperature (24 °C) were used as controls (CKs), and the other conditions were the same as those in the laboratory. After treatment, the fleshy stems of the plants were quickly frozen in liquid nitrogen and then stored in a −80 °C freezer until future use. After RNA extraction and quality control of the samples, libraries were constructed and sequenced via the next-generation high-throughput sequencing platform. We uploaded the raw transcriptome data to the National Center for Biotechnology Information database (NCBI, https://www.ncbi.nlm.nih.gov/) for storage, and the accession number was PRJNA1308681.

### 4.2. Identification of the Hsp70 Gene in H. undatus

The genome data, protein sequences, and annotation files (Gff3) of *H. undatus* were obtained from PGMD (http://www.pitayagenomic.com/), TAIR (https://www.arabidopsis.org/), and the protein sequence information of the Arabidopsis Hsp70 gene [27] was downloaded. In the first step, based on the protein information of AtHsp70, we used Blast-2.13.0 software to search for candidate proteins of the HuHsp70 gene family members in the *H. undatus* genome; in the second step, the Pfam database (http://pfam-legacy.xfam.org/ was obtained from Hsp70. After the gene-specific protein domain (PF00012) was analyzed, the sequences obtained in the above steps were retrieved via HMMER-3.0 software to construct a hidden Markov model, and the sequences that did not contain the structural domain were excluded. Finally, the online tools NCBI-CDD (https://www.ncbi.nlm.nih.gov/Structure/bwrpsb/bwrpsb.cgi, accessed on 9 January 2026) and SMART (http://smart.embl-heidelberg.de/) were used to remove the sequences without the Hsp70 domain or the incomplete domain to obtain the members of the *H. undatus* Hsp70 gene family and rename them according to their locations on the chromosome. The physicochemical properties, such as length, relative molecular weight, theoretical isoelectric point, hydrophilicity, and other physicochemical properties, of the proteins encoded by the HuHsp70 gene family were determined via the ExPASy website (https://web.expasy.org/). Additionally, the Plant-mPLoc online program (http://www.csbio.sjtu.edu.cn/bioinf/plant-multi/, accessed on 9 January 2026) was used to predict the subcellular localization of HuHsp70. In the above processes, an E value < 1 × 10^−5^ was assumed by default.

### 4.3. Sequence Alignment and Phylogenetic Analysis

To better elucidate the evolutionary relationships of *H. undatus* and other plant Hsp70 gene family members, we also used *Arabidopsis* (18) and rice (32) to construct a phylogenetic tree. We used the online version of MAFFT (v.7.526) for sequence alignment, and all parameters were the default values. Submit the compared results to trimAl (https://vicfero.github.io/trimal/, accessed on 9 January 2026) for pruning. Finally, iqtree (v.1.6.12), which is a beautification tool that uses the ChiPlot website (https://www.chiplot.online/), was used to construct a phylogenetic tree (the bootstrap value is 1000 repeats) https://www.chiplot.online/. The core principle for subfamily classification in this study is based on the phylogenetic tree topology derived from protein sequences, combined with gene structure (number and distribution of exons/introns) and conserved motifs to form a comprehensive determination.

### 4.4. Gene Structure and Conserved Motif Analysis

We can obtain information about the structure of the *HuHsp70* gene by using genomic data and annotation files. To identify conserved motifs, the MEME online tool (https://meme-suite.org/meme/, accessed on 9 January 2026) was used to retrieve possible motifs in the *HuHsp70* gene. The number of retrieved motifs was 10, and the other parameters were set to the default values. CFVisual software was used to visualize the above results [42].

### 4.5. Cis-Acting Elements of Promoters

For analysis of *HuHsp70*, for the cis-acting elements in the genes, we used TBtools to extract the nucleotides 2000 bp upstream of each sequence as the promoter region [43]. Through plant care (https://bioinformatics.psb.ugent.be/webtools/plantcare/html/, accessed on 9 January 2026), the cis-acting elements contained in the genes were predicted and visualized via ChiPlot after statistical analysis.

### 4.6. Chromosomal Positioning, Gene Replication, and Collinearity Analysis

To determine the location of the *HuHsp70* gene on the chromosome, we used TBtools software to generate the chromosomal location of *HuHsp70* based on the annotation files and sequence ID of *H. undatus* [43]. First, the chromosome length, gene location, gene density, and other information on the *HuHsp70* gene were extracted, and the intraspecific collinearity of the Hsp70 gene was determined via the Advanced Circles program. Simultaneously, family members with gene duplication events were screened, and then Ka, Ks, and Ka/Ks were calculated. The calculation formula for the differentiation time of the duplicated gene pair was as follows: if T > 1, it is considered to be affected by positive selection pressure; if T = 1, it is considered to be affected by neutral selection pressure; and if T < 1, it is considered to be affected by purifying selection pressure [44]. We chose four common fruits (apple, grape, sweet orange, and banana), and the collinear relationships among different species were analyzed via the MCSanX function.

### 4.7. RNA-Seq Analysis and Functional Annotation

To avoid problems such as low-quality sequences and adapter contamination in the transcriptome data, we performed data quality control (QC) on the raw data to ensure that the Q value of all the reads was greater than 30 and reference genome alignment rate > 92%. The reference genome (*H. undatus*) was downloaded from PGMD, and the filtered clean reads were sequence-aligned with the designated reference genome using HISAT2. Then, based on the alignment results and the position information of the gene on the reference genome, the number of reads for each gene was counted. The number of fragments for a transcript is related to the amount of sequencing data (or mapped data), the length of the transcript, and the expression level of the transcript. To allow the number of fragments to truly reflect the expression level of the transcript, we calculated the number of mapped reads and the transcription level in the sample. This length was normalized. This study used fragments per kilobase of transcript per million fragments mapped (FPKM) as an indicator to measure the expression level of transcripts or genes. We used featureCounts to calculate the value, and genes with FPKM values greater than 0.1 were considered expressed genes. Based on the unnormalized read count data of genes, we used DESeq2 to screen differentially expressed genes (DEGs). After variance analysis, the Benjamini-Hochberg method was used to calibrate the *p* value for multiple hypothesis testing to obtain the false discovery rate (FDR). The screening conditions for DEGs were a log 2 fold change| > or =1 and an FDR < 0.05. Finally, based on the log 2 (FPKM + 1) value, we used the R package (2e) to construct a heatmap of the Hsp70 gene expression profile of *H. undatus*.

Through the EggNOG-MAPPER database (https://github.com/eggnogdb/eggnog-mapper, accessed on 9 January 2026) of *HuHsp70*, Gene Ontology function (GO, https://geneontology.org/), and Kyoto Encyclopedia of Genes and Genomes (KEGG, https://www.genome.jp/kegg/, accessed on 9 January 2026) pathway, the annotation results were analyzed using TBtools. Finally, the online tool ChiPlot (https://www.chiplot.online/) was used to visualize the results [45,46].

### 4.8. Quantitative Real-Time PCR Analysis

To validate the accuracy of the RNA-Seq data, we used quantitative real-time PCR (qRT-PCR) to validate the expression trends of five randomly selected *Hsp70* genes, namely, *HuHsp70-1*, *HuHsp70-5*, *HuHsp70-11*, *HuHsp70-21*, and *HuHsp70-27*. First, the CDS of the target gene was extracted based on the gene ID. Gene-specific primers were designed via Primer 5.0. The sequences of all primer pairs are shown in Table S1. Next, the total RNA from all the samples was extracted via the Plant Tomospheric RNA Extraction Kit (Kangwei Century) according to the manufacturer’s instructions. cDNA was synthesized via MonScript™ RTIII All-in-One Mix with dsDNase (Monad) and a QuantiNova SYBR Green PCR Kit (QIAGEN), and qRT-PCR was performed according to previously reported procedures [28]. CrActin was selected as an internal reference gene to normalize the data. Three biological replicates were used for each gene, and the relative expression levels of the genes were calculated via the 2^−ΔΔCt^ method.

## 5. Conclusions

This study identified 27 *HuHsp70* genes from the complete genome of *H. undatus*. The physicochemical properties, phylogenetic relationships, gene structures, conserved motifs, cis-acting elements, chromosome distributions, and gene duplication events of these genes were analyzed, and their expression patterns were characterized to understand the functions of HuHsp70 family members. These genes play crucial roles in *H. undatus* growth, development, and stress response. Notably, our data suggest a potential mechanism in which the *Hsp70* gene may scavenge excess ROS and enhance the permeability of the cell membrane to regulate HS. These findings provide a valuable theoretical basis for elucidating the molecular mechanisms underlying the exposure of the Hsp70 gene to HS.

## Data Availability

All the data generated or analyzed during this study are included in this published article. The raw transcriptome data were uploaded to the National Center for Biotechnology Information database for storage, and the accession number was PRJNA1308681.

## Supplementary Materials

The following supporting information can be downloaded at: https://www.mdpi.com/article/10.3390/ijms27020816/s1.

## Abbreviations

The following abbreviations are used in this manuscript:

CAM	crassulacean acid metabolism
DEGs	differentially expressed genes
GO	Gene Ontology
FPKM	fragments per kilobase of transcript per million fragments mapped
FDR	false discovery rate
Hse	heat shock elements
Hsp	heat shock protein
Hsf	heat shock transcription factor
HS	heat stress
HT	high temperatures
KEGG	Kyoto Encyclopedia of Genes and Genomes
Mya	million years ago
NBD	nucleotide-binding domain
NCBI	National Center for Biotechnology Information
PBD	peptide-binding domain
QC	quality control
PGMD	Pitaya Genome and Multiomics Database
qRT-PCR	quantitative real-time PCR
ROS	reactive oxygen species
SBD	substrate-binding domain
