# Supplementary material for: Genome-Wide Identification and Expression Analysis of the Hsp70 Gene Family in Hylocereus undatus Seedlings Under Heat Shock Stress
Source: Int J Mol Sci. 2026 Jan 14;27(2):816. doi: 10.3390/ijms27020816 (PMC12840715; doi:10.3390/ijms27020816)
Supplement: Supplementary file 1 [file ijms-27-00816-s001.zip › ijms-4066771-supplementary.pdf]

Supplementary Materials

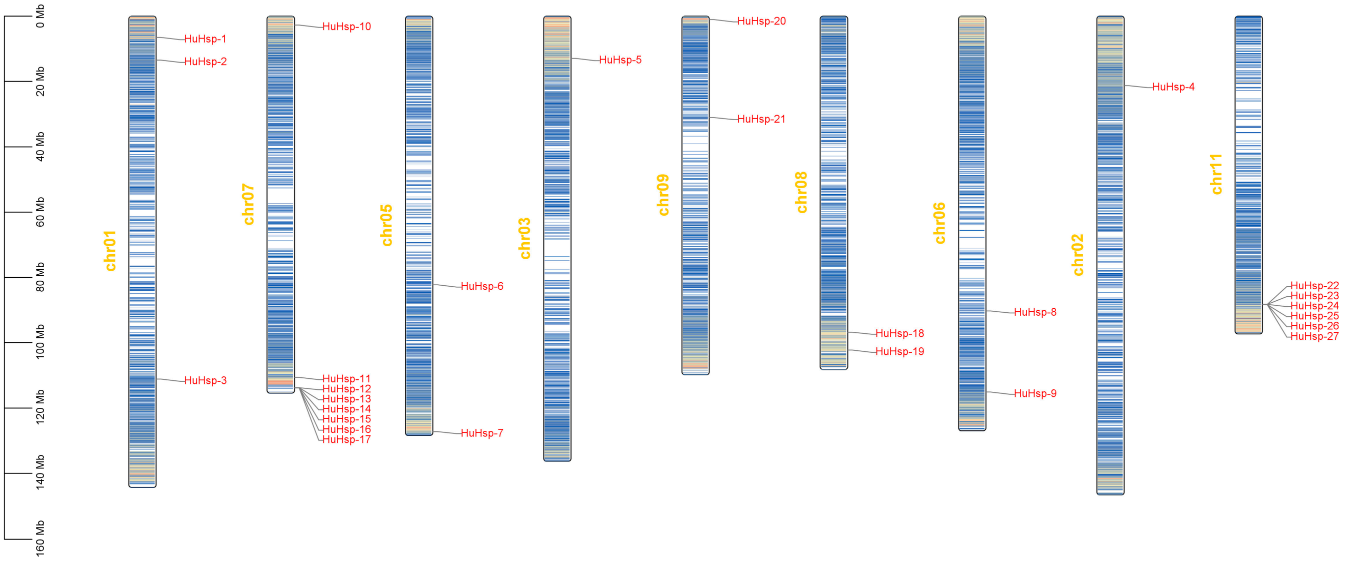

Figure S1. Chromosomal location of the HuHsp gene in *H. undatus;*.

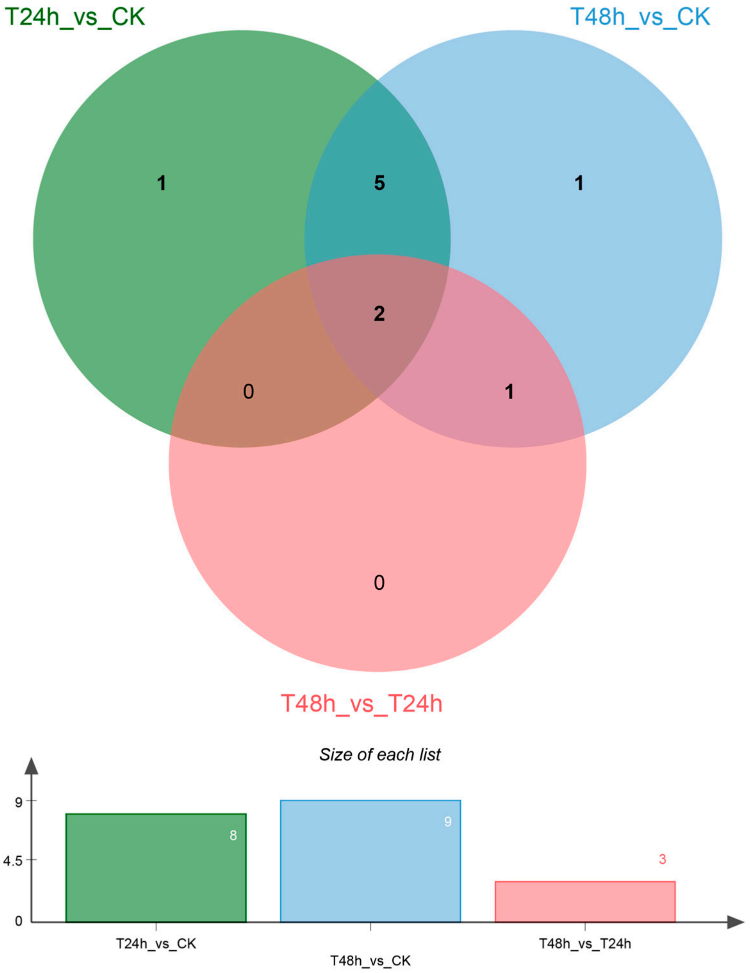

Figure S2. Wayne plots for heat treatment for 24 hours, 48 hours, and the control.

Table S1. Sequences of primers used for qRT–PCR;.

Table S1 Sequences of Primers used for qRT-PCR			
Gene		Sequence（5'-3'）	Product size
HuHsp70-1	F	TTCAGGAGGTAGTCCAGGCAATC	142bp
	R	GGGGTAACATCCAAAAGAAGCAA	
HuHsp70-5	F	GGGAAGGAGCCAACCAAGG	141bp
	R	GCCCAAACTCAAAGGAATCACAT	
HuHsp70-11	F	TGAGTGGCGATGCTAATGAGAAG	129bp
	R	TGGTGGGGATGGTGGAGTTT	
HuHsp70-21	F	TCAACCCTGATGAAGTAGTGGCA	109bp
	R	AACCCCAAGGACAATGGAGTAAC	
HuHsp70-27	F	CGCATCCCCAAGATTCAGC	161bp
	R	ATAAGCACAGCATGTCCATTACCA	
Actin	F	TCCAAAGGCTAACAGGGAGAA	114bp
	R	GTAGTACGACCACTGGCGTAAAG	

Table S2. Basic information and results of subcellular localization analysis of *HuHsp70s*;.

Table S2 Basic information and results of subcellular localization analysis of HuHsp70s							
Sequence ID	Number of Amino Acid	Molecular Weight	Theoretical pI	Instability Index	Aliphatic Index	Grand Average of Hydropathicity	Subcellular localisation
HuHsp70-1	676	72559.19	5.68	41.2	86.89	-0.307	mito
HuHsp70-2	642	73053.24	5.39	47.23	82.52	-0.423	cyto
HuHsp70-3	649	71161.6	5.09	32.91	83.27	-0.414	cyto
HuHsp70-4	651	71269.71	5.19	33.21	80.65	-0.439	cyto
HuHsp70-5	659	73439.67	5.21	34.92	88.48	-0.434	vacu
HuHsp70-6	702	75153.82	5.26	34.9	84.57	-0.334	chlo
HuHsp70-7	680	73021.91	6.02	37.8	86.82	-0.282	mito
HuHsp70-8	649	71161.6	5.09	32.91	83.27	-0.414	cyto
HuHsp70-9	635	68453.08	5.44	36.4	102.35	0.19	pero
HuHsp70-10	659	72231.2	5.28	35	83.22	-0.413	cyto
HuHsp70-11	647	71042.62	5.63	32.55	84.76	-0.412	cyto
HuHsp70-12	641	70891.86	5.92	35.57	88.1	-0.263	cysk
HuHsp70-13	630	69713.07	7.07	27.31	90.05	-0.273	plas
HuHsp70-14	646	71384.45	6.04	33.58	84.55	-0.313	cyto
HuHsp70-15	641	70890.92	6.07	35.05	88.1	-0.264	cysk
HuHsp70-16	607	67102.85	6.52	27.76	88.17	-0.288	plas
HuHsp70-17	646	71347.3	5.87	33.01	84.55	-0.311	cyto
HuHsp70-18	655	72995.86	5.1	33.5	86.64	-0.486	cyto
HuHsp70-19	699	75314.69	5.72	49.72	83	-0.326	nucl
HuHsp70-20	667	73556.43	5.17	32.22	86.57	-0.449	cyto
HuHsp70-21	697	74932.87	5.39	28.94	86.76	-0.316	chlo
HuHsp70-22	640	71145.62	8.58	37.06	85.33	-0.336	cyto
HuHsp70-23	641	71641.34	5.52	35.33	83.51	-0.389	cyto
HuHsp70-24	646	71285.17	5.78	32.69	85.15	-0.303	cyto
HuHsp70-25	646	71842.83	8.12	28.13	90.7	-0.229	plas
HuHsp70-26	641	70850.76	5.78	35.34	88.1	-0.262	cysk
HuHsp70-27	646	71130	5.86	30.02	85.31	-0.293	cysk

Table S3. Detailed information on the HuHsp70 gene family Motif 1~ Motif 10;.

Table S3 Detailed information on the HuHsp70 gene family Motif1~ Motif10	
Motif Number	Sequence
Motif1	VKNAVITVPAYFNDSQRQATKDAGIIAGLNVLRIINEPTAAAIAYGLEKK
Motif2	HLGGEDFDNRMVNHFVAEFKRKHNKDISGSPRALGRLRAACERAKRTLSS
Motif3	LIGEAAKNQAAMNPTNTIFDAKRLIGRRFND
Motif4	KELCKSINPDEAVAYGAAVQAAILS
Motif5	TRARFEELNMDLFRKCMEPVEKCLKDAKMDKSDIHDVVLVGGSTRIPKVQ
Motif6	VYEGERPIAKDNNLLGKFSLSGIPPAPRGVPQIDVCFDIDABGILNVSAE
Motif7	QDLLLLDVTPLSLGIETLGGVMTVLIPRNTTIPTKK
Motif8	SGRLSKEEIERMVKEAEKFKAEDEERKKASKAKNNLENYVY
Motif9	NDRVEIIANDQGNRTTPSWVAFT
Motif10	KNVLVFDLGGGTFDVSLLTIEKGVFEVKA

Table S4. Ka, Ks, and Ka/Ks calculations and divergence times of the duplicated *HuHsp70* gene pairs;.

Table S4 Ka, Ks, and Ka/Ks calculation and divergent time of the duplicated *HuHsp70* gene pairs					
Duplicated gene pairs	Ka	Ks	Ka/Ks	Purify selection	Time (Mya)
HuHsp70-10/HuHsp70-27	0.256441628	1.165806577	0.219969275	Yes	89.68
HuHsp70-8/HuHsp70-6	0.500935432	2.331613154	0.214845002	Yes	179.35
HuHsp70-5/HuHsp70-18	0.057628337	0.925264276	0.06228311	Yes	71.17
HuHsp70-4/HuHsp70-8	0.043414634	1.599264679	0.027146622	Yes	123.02
HuHsp70-2/HuHsp70-1	0.738110119	3.538521627	0.208592796	Yes	272.19

Table S5. FPKM expression levels of the *HuHsp70* gene in response to HS stress;.

Table S5 FPKM expression levels of the HuHsp70 gene in response to HS stress									
Gene name	CK-1	CK-2	CK-3	T24h-1	T24h-2	T24h-3	T48h-1	T48h-2	T48h-3
HuHsp70-1	24.8955	41.6829	38.964	49.1153	53.7162	35.9099	138.1375	202.5674	189.0972
HuHsp70-2	80.3448	83.7119	83.3303	145.4041	148.6324	121.1095	135.0797	141.2665	121.313
HuHsp70-3	0.6571	0.712	0.6716	0.3297	0.4485	0.3751	0.5716	0.5278	0.8566
HuHsp70-4	887.3632	718.162	778.0395	755.8028	795.8308	707.562	838.1823	803.1854	829.9041
HuHsp70-5	0.4457	0.2656	0.2412	6.973	8.5477	7.0782	10.627	12.2294	8.6476
HuHsp70-6	427.2336	384.5172	388.7825	659.5971	685.021	618.9525	660.0697	614.6404	496.9606
HuHsp70-7	60.8508	39.0849	46.8402	159.727	180.1842	150.5256	157.7939	142.8435	134.3117
HuHsp70-8	16.4737	43.5438	18.278	16.1069	12.5499	19.5946	12.397	14.4512	16.5219
HuHsp70-9	15.252	15.9609	19.11	39.463	44.1551	35.0934	29.331	28.783	25.5132
HuHsp70-10	21.7601	13.2759	28.8967	258.401	326.4781	231.6011	237.9514	243.324	235.2907
HuHsp70-11	11.6696	9.1494	11.7905	29.8826	27.1803	15.6673	45.525	47.3441	55.5715
HuHsp70-12	0.0521	0.3122	0.0533	0.0785	0.2593	0.23	0.1361	0.2563	0.1881
HuHsp70-13	0	0	0	0	0	0	0	0	0
HuHsp70-14	2.3388	9.3978	9.6553	9.7803	8.5904	13.7563	9.254	13.9516	8.3664
HuHsp70-15	0.4753	0.7053	0.7556	0.6095	0.4085	0.396	0.6201	0.7211	0.5715
HuHsp70-16	0	0	0	0	0	0	0	0	0
HuHsp70-17	0.3981	1.3444	1.953	1.6248	1.4079	2.0605	1.362	2.4933	2.0243
HuHsp70-18	3.5038	4.7694	6.0562	43.1023	48.8577	58.5288	50.3298	59.9725	69.0946
HuHsp70-19	0.6672	0.4318	0.8293	2.7688	3.2083	2.1954	1.835	2.5212	1.5983
HuHsp70-20	90.4729	155.0673	132.9582	64.5292	91.6475	147.1818	109.9561	149.9637	170.8148
HuHsp70-21	2.2719	1.6666	1.8289	10.8761	8.1101	9.153	4.6867	2.965	5.9475
HuHsp70-22	0	0	0	0	0	0	0	0	0
HuHsp70-23	0.2695	0.3972	0.3306	0.0812	0.1787	0.1189	0.0469	0.1178	0.0278
HuHsp70-24	1.3908	3.2516	4.1276	1.9326	3.606	4.2709	6.0484	6.0185	5.2377
HuHsp70-25	0	0	0	0	0	0	0	0	0
HuHsp70-26	1.8773	3.1219	3.7575	1.6747	3.6307	3.8876	3.107	3.6742	2.9562
HuHsp70-27	0.5274	3.1889	3.5387	1.7489	4.1558	5.9071	6.761	6.4623	4.6795

Table S6. GO enrichment details;.

Table S6 GO enrichment details		
Class	ID	Description
Molecular function	GO:0005515	protein binding
Molecular function	GO:0031072	heat shock protein binding
Molecular function	GO:0051787	misfolded protein binding
Molecular function	GO:0051082	unfolded protein binding
Molecular function	GO:0140662	ATP-dependent protein folding chaperone
Molecular function	GO:0044183	protein folding chaperone
Cellular component	GO:0005739	mitochondrion
Cellular component	GO:0005737	cytoplasm
Biological process	GO:0050896	response to stimulus
Biological process	GO:0042221	response to chemical
Biological process	GO:0009058	biosynthetic process
Biological process	GO:0006950	response to stress
Biological process	GO:0009266	response to temperature stimulus
Biological process	GO:0019538	protein metabolic process
Biological process	GO:0009408	response to heat
Biological process	GO:0061077	chaperone-mediated protein folding
Biological process	GO:0035966	response to topologically incorrect protein
Biological process	GO:0051604	protein maturation
Biological process	GO:0006457	protein folding
Biological process	GO:0006986	response to unfolded protein

Table S7. Physical and chemical properties and subcellular localization information of *HuHsp70-11* and *At1g16030*.

Table S7 Physical and chemical properties and subcellular localization information of HuHsp70-11 and At1g16030							
Gene name	Number of Amino Acid (aa)	Molecular Weight (Da)	Theoretical pI	Instability Index	Aliphatic Index	Grand Average of Hydropathicity	Subcellular Localization
At1g16030	646	70914.53	5.3	35.14	85.6	-0.377	Cyto
HuHsp70-11	647	71042.62	5.63	32.55	84.76	-0.412	Cyto
