# Supplementary figures and images for: Genome-Wide Identification and Expression Analysis of the Hsp70 Gene Family in Hylocereus undatus Seedlings Under Heat Shock Stress
Source: Int J Mol Sci. 2026 Jan 14;27(2):816. doi: 10.3390/ijms27020816 (PMC12840715; doi:10.3390/ijms27020816)

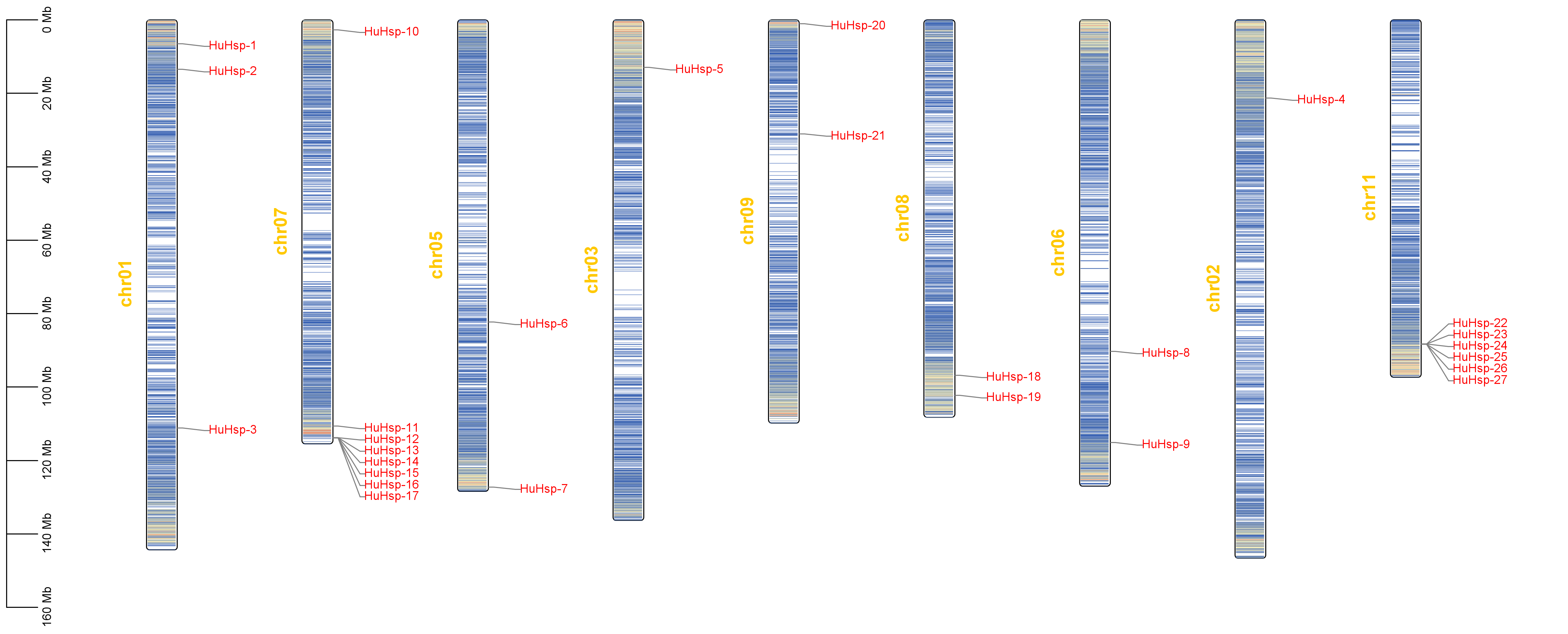

Supplement: Supplementary file 1 [file ijms-27-00816-s001.zip › Figure and Table S/Figure S1.tiff]

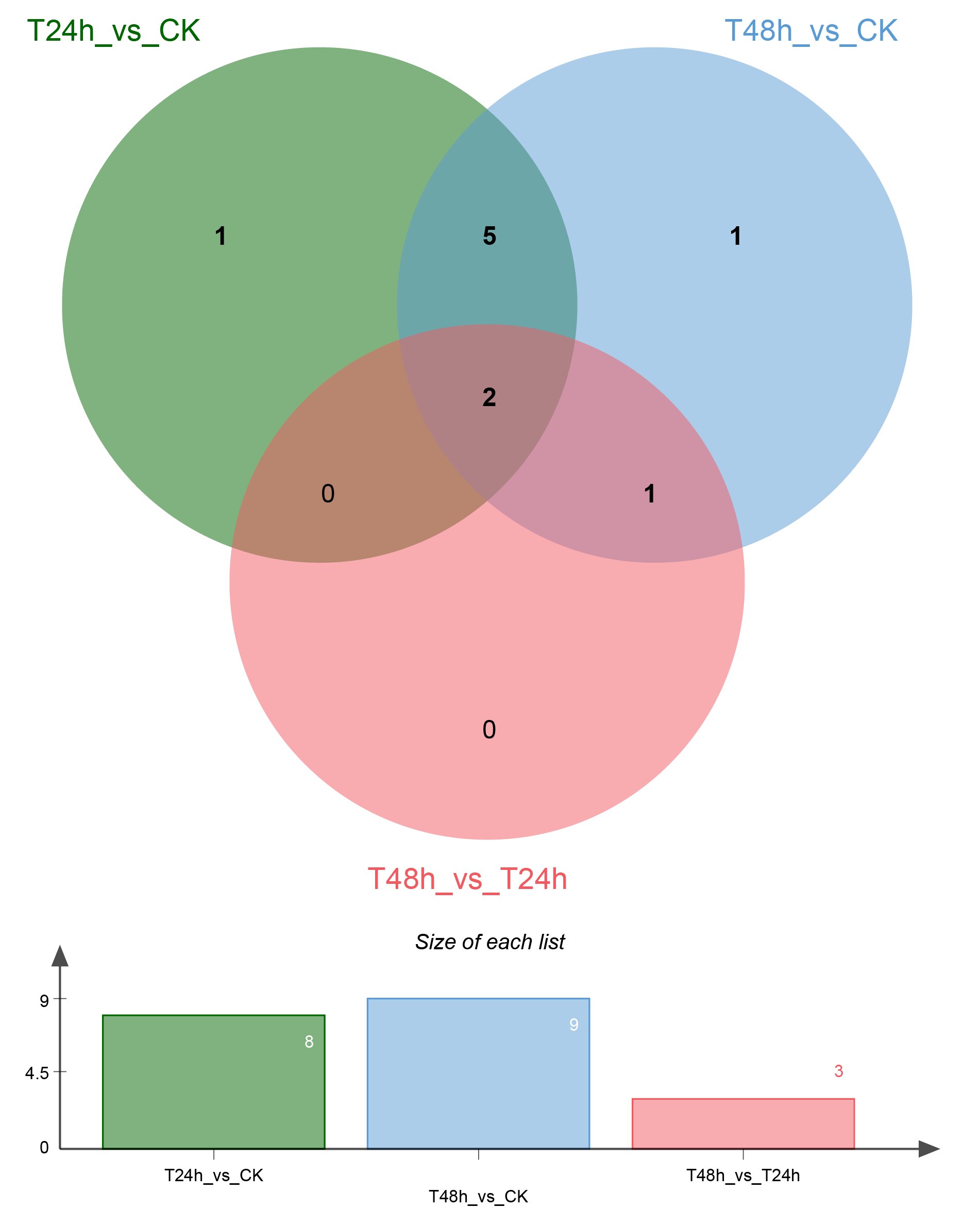

Supplement: Supplementary file 1 [file ijms-27-00816-s001.zip › Figure and Table S/Figure S2.jpg]

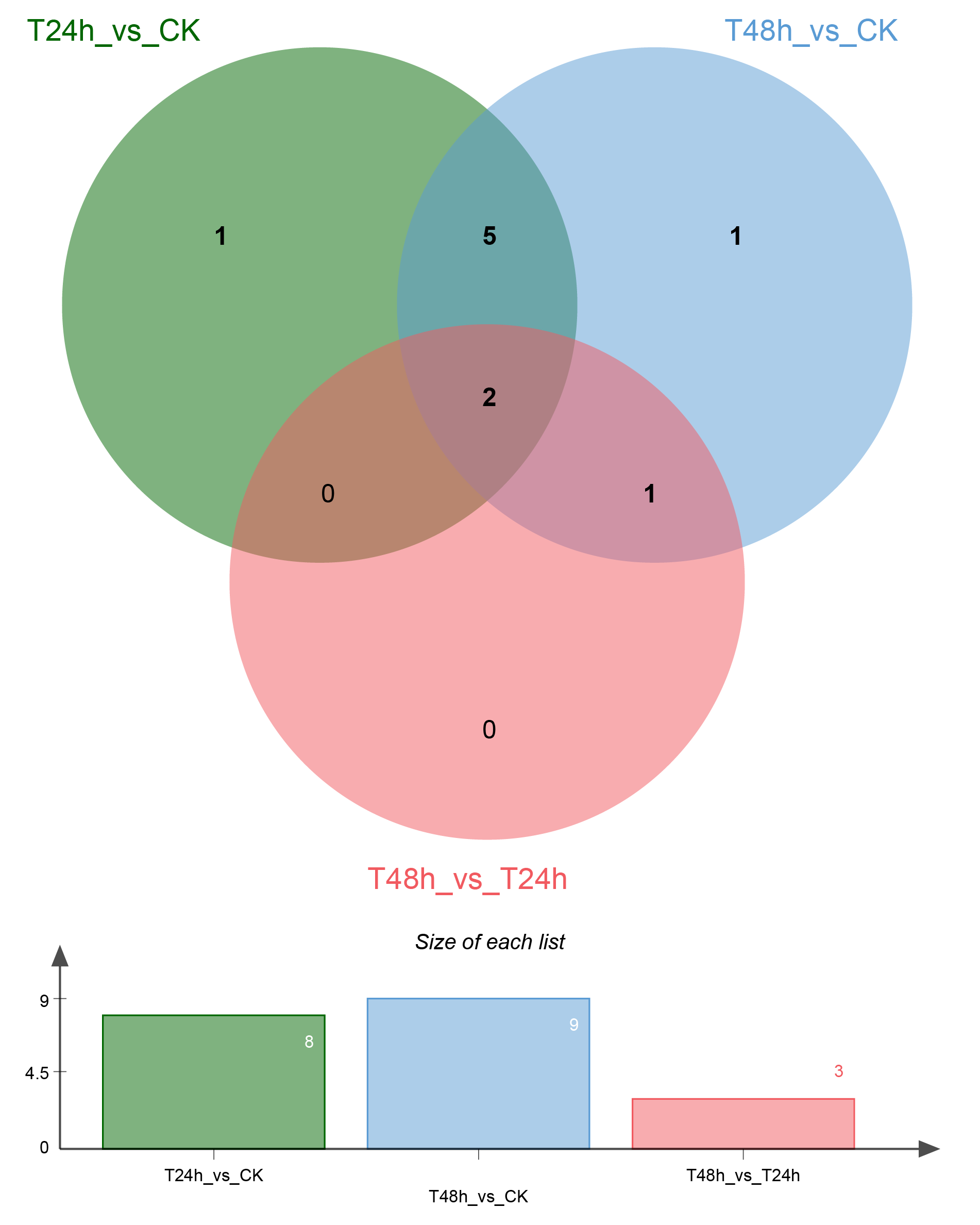

Supplement: Supplementary file 1 [file ijms-27-00816-s001.zip › Figure and Table S/Figure S2.tif]
